# Results of a New Method of Treating Whooping-Cough

**Published:** 1901-09-07

**Authors:** G. Arbour Stephens


### HOSPITAL CLINICS AND MEDICAL PROGRESS.

### RESULTS OF A NEW METHOD OF TREATING WHOOPING-COUGH.

By G. ARBOUR STEPHENS, M.D., B.S., B.Sc. Lond.

IN an article I published about two and a half years ago I advocated a new method of treating whooping-cough. This treatment consisted in, first of all, syringing both ears with a warm boracic solution, and then painting them with a 5-10 per cent. solution of cocaine in glycerine and water. Since that article was published I have had ample opportunities of testing the efficacy of the treatment, but, fearing lest a statement of my results might appear biassed, I have asked Dr. Trafford Mitchell, M.O.H., Gorseinon, for his permission to puolish the results obtained by him during a recent epidemic. The majority of the people living in his district are of the working class, the male population being employed in the local collieries or tinworks. His mode of carrying out the treatment was to syringe and paint the ears on his first visit, and after having demonstrated the process, to ask the parents to repeat it morning and evening. Such a method does not necessarily imply a very careful application of the treatment, and this must be taken into consideration when reading down the following lists. Dr. Mitchell agrees with me that there are three classes of children that do not react well to the treatment, viz.: (1) Those of a very nervous and irritable disposition; (2) those cutting their teeth; (3) those suffering from any local inflammation in the neighbourhood of the Eustachian tube. But even to these children the treatment sometimes brings relief if not a cure. These results are, I think, considerably better than any others obtained from internal medication, and there is the distinct advantage that if no benefit ensues, there is, at least, no depression such as follows the administration of belladonna.

| Sex. | Age. | Duration of Illness previous to Treatment. | Duration of Treatment. | Complications. | Remarks. |
|---|---|---|---|---|---|
| F | 2 years | 14 days | 8 days | Bronchitis | Whoop gone third day. Cough remained till eighth. |
| F | 4 years | 14 days | 8 days | Bronchitis | After second day whoop noticed only twice at night. |
| F | 8 years | 14 days | 8 days | .. | Change first day, slept better, whoop gone in five days. |
| M | 3 years | 12 days | 8 days | .. | Slept better first night. |
| F | 4 years | 5 days | 8 days | Otitis media | Three days before any difference. |
| F | 6 months | 5 days | 8 days | .. | Fifth day before any difference. Gone in eight days. |
| F | 6 years | 3 weeks | 6 days | | Whoop gone in four days. |
| F | 4 years | 2 weeks | 6 days | All bleeding from nose and mouth | Bleeding stopped in three days, whoop ditto. |
| F | 3 years | 2 weeks | 6 days | | Whoop stopped in three days. |
| F | 3 months | 10 days | 12 days | | Whoop only occasional on third day, but occasionally heard up till 24 days after, when child was cross. |
| F | 1 year | 6 weeks | 1 week | Subject to bronchitis | Much better in three days, whoop gone in one week but cough left. |
| M | 6 years | 3 weeks | 1 week | .. | Better in two days, but occasional whoop for one week. |
| F | 3 years | 2 weeks | 3 weeks | Bronchitis | Whoop gone in one week, but treatment continued while cough lasted. |
| F | 4 years | 3 weeks | 9 days | .. | .. |
| M | 3 years | 2 weeks | 8 days | .. | Cured. |
| M | 1½ years | 1 week | 10 days | .. | Cured. |
| M | 3 months | 3 weeks | 11 days | Bronchitis | Bronchitis left after whoop gone. |
| F | 2 years | 5 weeks | 8 days | Bronchitis | Whoop gone mostly on fourth, wholly on sixth. |
| F | 7 years | 2 weeks | 10 days | .. | |
| F | 3 years | 2 weeks | 3 weeks | .. | Marked difference in the week. Treatment continued three weeks for cough. |
| M | 3 years | 2 weeks | 1 week | .. | |
| M | 5 years | 2 weeks | 1 week | .. | Better in three days; whoop gone in one week. |
| M | 7 years | 2 weeks | 1 week | .. | |
| M | 4 years | 2 weeks | 2 weeks | .. | Greatly improved but not quite cured. |
| F | 2 years | 2 weeks | 2 weeks | .. | |
| M | 2½ years | 2½ months | 2 weeks | Bronchitis | Cured. |
| F | 8 years | At once | 1 month | .. | Modified. |
| F | 3½ years | At once | 1 month | .. | Very little effect. |
| F | 1 year | 3 weeks | 3 weeks | .. | Cured. |
| F | 2 years | 2 weeks | 3 weeks | .. | Cured. |
| M | 2 years | 2 weeks | 3 weeks | .. | Doubtful whether carried out properly. |
| F | 2 years | 2 weeks | 3 weeks | .. | Modified; better rest at night; not stopped. |
| F | 3 years | 2 weeks | 3 weeks | .. | |
| F | 1 year | 2/3 days | 6 weeks | Bronchitis | Greatly modified but not cured; when syringing stopped some evening or morning, paroxysms became severer. |
| M | 4 years | 2 weeks | 3 weeks | .. | |
| M | 5 weeks | 1 week | 3 weeks | .. | Modified, not cured. |
| F | 3 years | 1 week | 3 weeks | .. | |
| M | 8 months | 1 week | 3 weeks | .. | |
| M | 6 weeks | 1 month | 1 month | .. | Modified, not cured. |
| F | 2 years | 3 weeks | 1 month | .. | |
| M | 1 year | 8 days | 1 week | .. | Death from broncho-pneumonia. |
| M | 4 years | 2 weeks | 2 days | .. | Perforation of tympanic membrane rendering continuance of treatment impossible. Death from broncho-pneumonia. |

The following four cases were under the charge of Mr. A. Lloyd Jones, M.O.H., Oystermouth, Glamorgan:—

| Sex. | Age. | Duration of Illness previous to Treatment. | Duration of Treatment. | Complications. | Remarks. |
|---|---|---|---|---|---|
| F | 1 year | 5 days | 1 week | .. | Cured. |
| M | 10 years | 7 days | 3 weeks | Old otitis media gave rise to much pain in ears. | Whoop disappeared second week. The pain was relieved by the painting. A pharyngeal cough remained. |
| *M | 13 years | 7 days | 2 weeks | .. | Returned to school at end of three weeks. |
| *M | 13 years | 7 days | 2 weeks | .. | Returned to school at end of three weeks. The painting caused pain and weeping of the eyes. |

* These cases were isolated at a boarding school; their reurn at the end of three weeks gave rise to no other case.

